# Hospital and Institutional News

**Published:** 1916-04-15

**Authors:** 


### HOSPITAL AND INSTITUTIONAL NEWS.

### THE HORRORS OF WITTENBERG.

England and, we trust, the civilised world as well, is ringing with the horrors of the prison camp at Wittenberg, exposed in the official report of the Commission which investigates German brutalities and enormities. No wonder the Germans hesitated for many months to set free the witnesses who had seen such things: having committed such abominable crimes, the breach of the Geneva Convention involved in detaining medical officers for a year or two was nothing at all in comparison. Six English doctors, three only of whom have lived to tell the tale, went through the horrors of that Black Hole, the full details of which are infinitely more disgusting than anything in the annals of Calcutta, because the outrage was spread out over many weeks. The one incident which shows the German authorities exhibiting any sense of the fitness of things was the bestowal of the Iron Cross upon the chief surgeon, the man who contrived the whole devilish cruelty and shouted abuse from outside the barbed wire at the devoted heroes inside it. He and the conqueror of the *Lusitania* are a pair of worthies for whom no other decoration in the universe would rightly suffice.

### TWO MEDICAL OCTOGENARIANS.

During the past week two very old and greatly respected members of the medical profession have passed away. Sir Thomas B. Crosby, M.D., who was Lord Mayor of London in 1912, had already then passed his eightieth birthday, and was the first medical man in history to fill that distinguished office. He had been in practice in the City for the greater part of his active career. The usual baronetcy conferred upon the Lord Mayor was withheld in his case, owing to some criticisms of the party in power uttered by a guest at an official banquet: in the eyes of any but party politicians the incident would seem creditable rather than the reverse. Sir Alexander R. Simpson was killed by a motor-car in Edinburgh, aged eighty-one. A nephew of Sir J. Y. Simpson, he was professor of midwifery and gynæcology at Edinburgh University for thirty-five years. He was a highly religious man, and worked enthusiastically to promote the religious, as well as the professional, development of his students. He was knighted in 1906, the year after his resignation of his professorship.

### A TRIBUTE TO HOSPITAL TREATMENT.

The Report (for 1914) of the Medical Officer of Health for the County of London, which was published about the end of March, draws attention to certain peculiar differences between town and country rates of mortality disclosed by the Registrar-General's returns and to the explanation offered of these differences. Pneumonia and appendicitis are far less deadly in London than in rural areas. This curious fact can hardly be due to the factor of migration which disturbs the relative death-rate in respect of chronic diseases, such as consumption: for there is no time or opportunity for the patient stricken with pneumonia to leave the neighbourhood where he happens to find himself at the moment. The difference is ascribed by the Registrar-General to the influence of hospital treatment and careful nursing. That the modern hospital, seen at its best in our large towns, can actually influence mortality rates among the general population sufficiently to call for comment in official reports is a tribute than which it is difficult to conceive of anything more remarkable and arresting.

### TEAM WORK IN PLASTIC SURGERY.

In the current issue of the *Journal of the Royal Army Medical Corps* appears an article describing the remedy of certain deformities of the face, the joint work of an ophthalmic surgeon, an ear and throat surgeon, and an Associate of the Royal Academy! This is, indeed, what the Americans call team work; all three of the trio are members of the 3rd London General Hospital; the first two as officers, the third as a sergeant. Briefly, the surgeons did their best to restore a foundation of sound tissue in certain cases where a large amount of the face had been bodily removed by gun-shot wound; and when their efforts came to an end they called upon Sergeant Derwent Wood, A.R.A., who most ingeniously made artificial faces to mask the deformities. He takes a plaster mould of the face, gaping orifices being filled with cotton-wool and covered with gold-beater's skin. From this a clay or plasticine cast is taken. On this the sculptor models his reconstruction of the face, which in due course is reproduced by an electrotyper in virgin copper one-thirty-second of an inch in thickness. This is finally coated with silver, and finished off with a coat of cream-coloured bath enamel as a preparation for the final pigmentation in flesh tints. It is found that real hair for eyebrows and eyelashes will not stand the weather; the former are painted in, and split tinfoil supplies the latter. The mask is kept in place by spectacles which are adjusted at the requisite angle to give a well-distributed pull over the ears.

### AN ASSISTANT MEDICAL OFFICER'S PEERAGE.

The assistant medical officers at mental institutions have suffered much from discouragement, monotony, absence of opportunity for promotion or for marrying, and the like. It is therefore, we hope, symbolic that a peerage has fallen, to speak like a layman, on one of them. The event has occurred to Dr. Gervase Alexander, one of the assistant medical officers at Bodmin Asylum, who, we understand, when legal forms have been fulfilled, can take his place in the Upper House as Lord Cobham. In view of the fact that medicine is now practically unrepresented in the House of Lords, we hope that the new peer will be an active member. The peerage of Cobham, together with those of Burg and Strabolgi, have been for a very long period in abeyance, but the legal process by which that abeyance might be determined was begun by various claimants, including the late Dr. Alexander, of Blackwall Lodge, Halifax, whose recent death occurred only a few days before the decision to determine the abeyance was made known. The late Dr. Alexander, who had retired from practice, had formerly been senior physician to the Halifax Royal Infirmary, and was well known in his day, and even after his retirement from active professional work, as one of the early students of the open-air treatment, as a medical man of repute whose very hobbies were scientific, and whose leisure, for instance, was much occupied at one time with the study of that old and favourite problem—the factors which determine the nature of sex in the offspring. His eldest son, referred to above, entered on his father's profession, and has spent his time in institutional work, latterly at Bodmin. If ever the Assistant Mental Medical Officers' Association, which was formed, it will be remembered, not long before the war, needs a spokesman in high position, Dr. Alexander, when in a position, as Lord Cobham, to exercise his prerogative in the Upper House, should be able to render invaluable service not only to his former colleagues, but to the hospital world at large. Dr. Alexander is unmarried, and his younger brother is the present heir.

### A NEW MEDICAL JOURNAL.

Yet another name is to be added to the list of medical journals devoted to special subjects. It is comforting that in this particular instance the addition of one results in one less. The *Proctologist and Gastro-Enterologist* is the name of a new journal to be issued quarterly in the United States. Our new contemporary will be a fusion of the *Proctologist*, a journal dealing with diseases of the rectum, which commenced publication in 1907, and the *American Journal of Gastro-Enterology*, which was first published in 1911—a journal devoted to subjects pertaining to digestion. The editorial staff consists of well-known American authorities, Dr. R. W. Barnes, St. Louis, being responsible for proctology, Dr. A. Bassler, New York, and Dr. L. Brinton, Philadelphia, for gastro-enterology, and Dr. A. L. Benedict, of Buffalo, for dietetics.

### THE LATE COLONEL BARKER.

Among the latest casualty returns is the name of temporary Colonel A. E. J. Barker, A.M.S., who died on April 8 of pneumonia and nephritis while on active service, aged sixty-five years. Colonel Barker had been a distinguished member of the staff of University College Hospital for thirty years, and it will come as a surprise to many to hear that he was before that surgeon at the City Hospital, Dublin. He became Professor of Surgery at University College in 1893, and was best known professionally for his especial interest in the surgery of joints and in spinal anæsthesia: of the latter he was an enthusiastic supporter and a most able exponent. But he was in every sense of the word an all-round man, and was a surgeon thoroughly to be trusted in any branch of his science. Since November 1914 he had served as consulting surgeon to the Southern Command.

### "TOM BATES, SURGEON."

On Monday, April 3, there passed away in Worcester, after a brief illness, a man who was not only a notable surgeon, but a remarkable personality. Tom Bates received his professional training in Scotland, qualifying at Edinburgh in 1866. He further pursued his medical studies in Paris and Vienna, travelling for a great part of the way on foot. In 1879 he was appointed hon. surgeon to the Worcester General Infirmary, and often referred to the day of his election as the proudest in his life. He was unsparing of himself in his devotion to his profession, and having a very high standard of work for himself, was intolerant of anything suggesting "slackness" in others, and many will realise the debt of gratitude they owe him for this. He had a keen appreciation of art, poetry, literature, and nature, and was a man of wide culture. In 1909 he resigned his post of senior surgeon to the infirmary after thirty years of devoted service, and was appointed hon. consulting surgeon. In 1913 he almost entirely gave up practice, but still rarely missed an operating day at the infirmary, and on two occasions when that institution was in straits came back and acted as "house surgeon" for a few days. To enable both his sons to serve in the R.A.M.C., Mr. Bates returned to harness (at the age of seventy-two), and carried on the work of a large practice until March 30, when, after attending a case in the early morning in bitter weather, he succumbed to an attack of influenza and died after five days' illness. The funeral, which took place on the 7th, was marked by a simplicity and absence of conventionality which were in accordance with his whole life. The plate on the coffin was the door-plate which had been so familiar to all in Worcester: "T. Bates, Surgeon."

### THE UNIFORM SYSTEM AT NEWBURY.

The report of the managing committee of the Newbury District Hospital to the subscribers is chiefly of interest in its references to the reform in the system of accounting which has been adopted. It would appear that a year ago there was some dissatisfaction with the then existing system, and a finance sub-committee was asked to investigate. A firm of chartered accountants in Oxford were requested to report, and their examination disclosed that "owing to a lack of discrimination between capital and income in past years" the deficiency on the year's working (1914) was in reality £591, not £255, as shown in the balance-sheet. The firm in question also, it is said, prepared accounts and a balance-sheet which will serve as a model for the future. These are "practically the Revised Uniform System, adopted by King Edward's Fund and most of the leading London hospitals." As the year 1915 showed a surplus of £187 on income and expenditure account, the fees of this firm of accountants will be no great strain upon the institution; but surely, at this time of day, it is not necessary for country hospitals, large or small, to go to such expense merely to be told what everyone already knows about the Uniform System? Besides, there is an up-to-date edition of the "Uniform System of Accounts" now being printed, which contains specimen accounts for such institutions. It will be published shortly by The Scientific Press, Ltd., 28 and 29 Southampton Street, Strand, London, W.C.

### LEICESTER ROYAL INFIRMARY.

The manner in which the annual general meeting was organised on March 29, and the interest and publicity secured, admirably illustrates the value of this form of propaganda in advancing the prosperity of an important hospital. Reference has already been made to the annual report, but the salient features of the year's work were so well brought out by Colonel C. J. Bond, who moved the adoption of the report and accounts, that a further notice may be found useful. The infirmary had experienced the usual difficulty in balancing the claims of civil and military patients, and the net result in 1915 was that though 692 soldiers had been treated, only 165 fewer civil patients passed through the wards. When it was realised how large a number of men were away from the town it would be understood that the proportion of civil cases treated was, in relation to the requirements of the neighbourhood, quite a fair one. The *x*-ray department had been particularly busy during the year, owing largely to the military cases. The infirmary had experienced serious difficulties in obtaining necessary drugs and household commodities, and a compliment was paid to Mr. Harry Johnson, the house governor, who had anticipated many of the difficulties and taken necessary steps to meet them. Mention was made of the excellent work of the Saturday Hospital Society, whose collection for the year was £16,449, of which the infirmary received £11,000.

### BRISTOL'S RED CROSS RECORD.

A very sturdy local patriotism flourishes in Bristol and the vicinity, and the recently published reports of the City and County Branch of the British Red Cross Society show what an excellent thing such patriotism is and what splendid fruits it has brought forth. The basis of the organisation was provided by the Bristol Royal Infirmary, acting in conjunction with the local Red Cross branch, as long ago as the year 1911. On the outbreak of war not only was a first-class Base Hospital ready to hand, but the entire resources of the professional and voluntary medical services within the local area were mobilised. According to the report just issued, in the year 1915 no fewer than 11,773 sick and wounded soldiers were treated. Full statistics are provided in the report concerning the details of this immense and valuable work. Space precludes the mention of more than a few of these: thus ten ladies collected between them more than 150,000 eggs, and, all told, 176,000 were distributed to the institutions where the soldiers are cared for. Over £17,000 was subscribed, without any interference with the claims of the central organisation of the Red Cross in London; over £12,000 was expended, and the remainder was carried over to help meet the needs of 1916. Much clothing has been provided, and many comforts—including over a million cigarettes. Twenty-three haunches of venison is another item in this list. Altogether a very notable achievement, and a feather in Bristol's cap.

### A STRIKING TESTIMONIAL.

At the annual general meeting of the Royal National Hospital for Consumption, Ventnor, the Chairman (Mr. P. B. Burgoyne) invited Mr. Lyons to address the meeting. Mr. Lyons explained that nine years previously he had been a patient at the hospital for several months: that he had gained nearly three stone in weight there, and maintained that gain since: that the skill and kindness of the medical staff and of the nursing staff were then all that could possibly be desired; and, in fact, that the whole institution was admirably conducted and managed. He himself was in such robust health that he had attested for the Army, and been passed as sound. In view of the opinions that have been expressed concerning the enlistment of men who have at some previous time suffered from pulmonary tuberculosis, another of his reminiscences was of some interest. A soldier friend of his was about to be discharged from the Army for tuberculosis, when Mr. Lyons suggested that he should be treated at Ventnor. This course was adopted; the disease was arrested, the patient went back to his regiment, and has been through the great campaign from the first day to the present. One swallow does not, of course, make a summer; and active service has unquestionably resuscitated a quiescent tuberculosis in many cases. But it is interesting to know that this is not a universal occurrence nor an inevitable one.

### V.A.D. NURSES AND THE WORCESTER INFIRMARY

The annual meeting of the subscribers to the Worcester Infirmary was notable for two principal points. First, a rousing speech from the Mayor (Alderman H. A. Leicester), in which the plain facts about the financial situation were stated with force and courage; and, secondly, an equally interesting speech from the Dean of Worcester, showing how the institution has gained in general reputation through opening its doors to Red Cross nurses. The Mayor pointed out that the town has a population of 50,000, and that the infirmary has a subscription list of but £800. Further, there was a deficiency on last year's working of nearly £700: the facts speak for themselves, and after the Mayor's timely reminder we look to the people of Worcester to wipe out the slur they suggest. The Dean was equally frank: he said he had been elected to the management committee by the wishes of some dissatisfied persons unnamed. After two years' experience, during which he has looked into matters for himself very closely, he is convinced that the institution is "thoroughly well managed." He also bears testimony that the V.A.D. workers, who have been in the infirmary in large numbers, have done much to engender greater confidence in the institution, because they had expressed favourable opinions which had swamped "tittle-tattle" of the reverse description. There are many institutions which only require to be better known to be more appreciated: possibly many others have benefited in like manner through the influx of V.A.D. workers.

### THE HOSPITAL STANDARD AT WORCESTER.

It is not clear from the Dean of Worcester's guarded remark, quoted above, whether he is fully alive to the existence of what has come to be known as "the hospital standard." It is this, in any case, which must be achieved, and the fact that a body of Worcester citizens were actively interested enough to ask him in an official capacity to investigate is an encouraging sign. But the hospital standard is not to be achieved, as it were, by deputy. The people of Worcester, town and county, must realise that the infirmary is what they care to make it, and the only way to make it a first-class hospital is to make it a living centre of interest in their own lives.

### CARNARVON INFIRMARY.

Like many other similar institutions up and down the country the infirmary of the Carnarvon Board of Guardians has been turned into a military hospital, and £464 expended by the Guardians on the fitting up of the place for the reception of the soldiers has been refunded by the War Office. For the past month a number of convalescent soldiers have been in occupation, and it is anticipated that by the end of June the number will have increased to about a hundred, including those accommodated in tents. The military representative who has inspected the infirmary pronounced it the best situated and best adapted for this particular purpose of any he had seen in the country. The Local Government Board has sanctioned the payment to Dr. Robert Parry, the workhouse medical officer, of an annual salary of £310 conditionally upon his taking sole charge of the military hospital, the salary to be paid by the War Office.

### TO CLOSE OR NOT TO CLOSE.

The Kensington and Fulham General Hospital has, during recent years, several times been on the point of closing its wards, if it has not actually done so. The district recently has had another close shave of losing valuable hospital accommodation, and financial difficulties were so acute, as was reported at the annual court of governors held on March 24, that steps had been taken to give effect to a resolution of the board that the whole of the work of the in-patient department should be suspended. The services of most of the nurses were dispensed with, and the beds were gradually closed down until only four remained. Fortunately, two large donations turned the tide and, though the children's ward is still out of use, the adult beds have been reopened. These crises bear hardly upon the officers of the hospital, the institution itself, and the poor of the district. A hospital is a machine made up of many intricate parts, and abruptly to cut off an important part of its activities is a serious matter, breaking, as it does, continuity of service on the part of medical, nursing, and general staff, a factor in successful hospital administration which must not be interfered with except for the gravest reason. Lord Claud Hamilton, the President, who took the chair at the annual meeting, backed his opinion of the genuineness of the needs of the institution when he gave a substantial donation towards present wants; that others will in a similar way signify their opinion of the utility of the work of the Kensington General Hospital is the wish of all friends of the institution.

### WAR NOMENCLATURE OF WARDS.

There is a distinctly martial sound about the names "Kitchener" and "Cannon," adopted for the two wards added to the Bolton Infirmary for the reception of the institution's share of wounded soldiers. The infirmary has, consistent with the many other claims upon its resources, played the part that might be expected of it in helping the Army Medical Department through its difficulties both as regards the reception of the wounded and the provision of medical officers for service with the Forces. Medical women at Bolton, as happened so often elsewhere, have stepped into the breach, and the three house surgeons' posts have all been ably filled by them. The financial position of the infirmary is, temporarily we hope, rather unsatisfactory, as another four-figure deficit brings the total indebtedness up to £2,722. The committee's report states with some apprehension that this sum must probably come out of accumulated funds, and expresses the opinion that the strengthening of the reserve is more important than hitherto, as the prospect of State aid for hospitals, in view of the present heavy national expenditure, may be regarded as remote. This reason for saving is interesting; and, while in no way wishing to discourage a commendable effort to build up an endowment, it may be said that it is a very debatable point whether large aggregate invested funds might not be an inducement to legislators favourable to State aid to push forward their ideas on the grounds that, after all, the change from voluntaryism would not immediately plunge too deeply into the pocket of the taxpayer.

### WAR'S EFFECT ON WOMEN.

No one could have a better opportunity of judging the physical and mental effects of present unusual social conditions upon women than the hospital physician. This point was well brought out at the annual meeting on March 29 of the Birmingham and Midland Hospital for Women. Mrs. Feeney, who presided, said that the condition of many of the patients had been induced by anxiety, the result of the war. Mr. H. Martin, who seconded the adoption of the report, said that in Birmingham there was perhaps as much as anywhere an unprecedented period of overstrain, both physical and mental. Hitherto men had borne the greater part of the struggle of life, but as they were being drafted away from the ranks of employment the ever-widening gaps thus formed had to be filled by women, many of them unfitted for the task owing to lack of training. On the mental side they had anxiety and a sense of dread of loss, and these harassing conditions were chiefly responsible for the present high rate of breakdown among women.

### MANCHESTER DOCTORS AND THE WAR.

Some interesting statistics are forthcoming illustrating the splendid response made by Manchester medical men to the nation's call. Being a hospital city and the centre of a medical college, it has been able to release a very large number of men. Roughly speaking, there are 540 doctors on the Manchester register, including public officials, house surgeons, and assistants, and already 130 of these are on active service in the field, while eighty are engaged at the 2nd Western General Hospital in Whitworth Street, Manchester. There are thus left 350 to draw upon, but the greater number of these are above Army age. Quite apart from those assisting at the numerous Red Cross hospitals in the district, 90 per cent. of the remaining eligibles —who number 140—have enrolled for service when required. In many parts of Manchester however, the maximum number of doctors have been withdrawn, and it is felt that not more than an additional fifty can now be taken, except at the expense of the civilian population. Hospital doctors were among the first to offer their services to the military, and of the 130 medical men from Manchester now actually in the Army, only fifty were general practitioners.

### POOR-LAW MEDICAL OFFICERS IN WAR HOSPITALS.

An unusual position appears to exist in the case of one of the medical officers of the Devonport Board of Guardians. The Ford Hospital belonging to them has been taken over by the military authorities as a war hospital. At a recent meeting of the Guardians it was said that the Guardians' medical officer of the institution was receiving both his military pay and his Poor-Law salary, and that the arrangement had received the sanction of the Local Government Board. If the facts are as stated, this would appear to be in direct contravention of the general ruling of the Local Government Board, that under such circumstances the medical officer is to receive either his military or his Poor-Law pay, whichever is the larger. The fairness of this ruling cannot be gainsaid, and unless there are exceptional circumstances in the case under discussion, the Guardians should approach the Local Government Board again and ask for a reconsideration of the matter.

### PAYMENT FOR MEDICAL ATTENDANCE ON SOLDIERS.

A very acrimonious discussion took place at the last meeting of the Belfast Board of Guardians upon the payment to be made to their medical officers for attendance upon the soldiers under treatment in the Poor-Law infirmary. The infirmary committee reported that the military authorities were paying one guinea weekly for each soldier under treatment, and that the cost of maintenance was 14s. 6d. They suggested that the remaining 6s. 6d. should be apportioned, 3s. 6d. to the visiting medical officers for each patient attended, and 3s. to the resident medical and other officers. The opponents of this suggestion pointed out that at the voluntary hospitals no payment was made to the visiting-staff, and contended that their own medical officers were guilty of an unpatriotic and contemptible act in asking payment for the services they were rendering. It is difficult to understand how statements of this kind can be put forward as serious arguments. Unfortunately, however, the Belfast Guardians are not alone in their views that the charity of the medical profession in war-time should be exploited to the fullest extent. But if just demands for payment by medical men are to be met with abuse on the grounds that much work is done by them gratuitously already, they may well wonder whether it may not be politic to demand payment for all services rendered to public institutions.

### A MEDICAL SUPERINTENDENT'S HONORARIUM.

The Camberwell Board of Guardians decided some months ago to grant to Dr. Keats, the medical superintendent of their infirmary, £75 for extra services rendered in connection with his additional duties under the National Insurance Act. The Guardians were desirous of increasing his salary £100, but the Local Government Board refused to sanction this sum for the year, but permitted the Guardians to pay Dr. Keats £50. Thereupon the Guardians, after several refusals from the Local Government Board to sanction the larger amount, took the matter into their own hands, and under the powers vested in them granted to Dr. Keats £75 on two separate occasions for his services in two consecutive years. There has, however, arisen a startling development to their action. Objection was taken at the public audit of the accounts by ratepayers to paying Dr. Keats these two sums of £75, and the Public Auditor has upheld their objection, surcharging the Guardians who signed the cheques the sum paid to Dr. Keats. Of course there will be an appeal to the Local Government Board for relief, and in all possibility it will be granted; but it shows that the Local Government Board will not allow its decisions to be overruled in this way by a Board of Guardians. It would have been much better for the Camberwell Board of Guardians to have tried again to soften the hearts of the officials of the Local Government Board rather than to have defied openly the ruling of the higher authority. It is apparent that the Guardians will not be able to continue to grant this £75 yearly in the same way, for, if they do, they may not expect any relief from Whitehall.

### MEDICAL STAFF CHANGES UNDER THE M.A.B.

The vacancy in the senior medical post at the North-Western Fever Hospital, caused by the resignation of Dr. J. MacCombie, has been filled by the appointment of Dr. E. W. Goodall, the medical superintendent of the Eastern Fever Hospital at Homerton. Dr. Goodall has been for years a superintendent, but the change from Hackney to the more salubrious neighbourhood of Hampstead may be regarded, in a sense, as promotion. The post of medical superintendent at the Eastern Hospital has been given to the senior assistant medical officer, Dr. J. H. Whitaker, M.D., D.P.H. He has acted as medical superintendent during Dr. Goodall's recent absences in France on war service, and has been a senior assistant medical officer for over fifteen years. He entered the service of the Metropolitan Asylums Board nearly twenty years ago. Another recent appointment is that of Dr. C. Price to the Children's Infirmary in Cleveland Street. He has been transferred from the Park Hospital, where he was assistant medical officer, and is to act temporarily as medical superintendent at this recently acquired institution for children. This infirmary formerly belonged to the Holborn Guardians, and was taken over by the Metropolitan Asylums Board, together with the stock of old furniture and some new linen and crockery, about three months ago.

### WOMEN DISPENSERS.

An interesting scheme for the training of women as dispensers is being tried at Leeds. The great demand there at present for women dispensers in connection with Health Insurance, as well as at the military hospitals, has led the Leeds Education Committee, in association with the Leeds Public Dispensary Board, to announce a course of instruction to prepare women for the Assistant Dispensers' Examination of the Society of Apothecaries (London). The curriculum will include materia medica, dispensing and practical pharmacy, taken at the Public Dispensary, and chemistry, taken at the Central Technical School. The course will occupy eight half-days a week and will continue for six months. Students must be over eighteen years of age, and as the number to be admitted is limited, preference will be given to those who have passed a recognised preliminary examination, such as the Oxford or Cambridge Local or Matriculation.

### WINDFALLS FOR BRIGHTON HOSPITALS.

The will of the late Miss Elizabeth Simpkins contains some notable bequests to institutions of her own town, Brighton. The Sussex County Hospital will receive £5,000; the Royal Alexandra Hospital for Children, Brighton, the Brighton and Hove branch of the Surgical Aid Society, the Throat and Ear Hospital, Brighton, the Eye Hospital, Brighton, the Asylum for the Blind, Brighton, and the Asylum for Deaf and Dumb Children, Brighton, are left £1,000 apiece. There are further charitable and other bequests, after which the residue of the estate is left for distribution by the trustees among the deserving poor of Brighton or among societies which are carrying out charitable work. The total value of the estate is about £41,000 gross.

### HOSPITALS AND THE MINERAL-WATER TAX.

The imposition of a tax on mineral waters promises to lead to a change in the practice of those hospitals which have in the past been accustomed to purchase aerated waters from outside makers. The large hospitals which already manufacture their own mineral waters will not be affected, for, while the Finance Bill (Clause 8) provides that a licence fee of five guineas must be paid by any person using any mechanical contrivance for making aerated water, Sub-Clause 3 of this clause exempts from the obligation of taking out such a licence persons who use "machines or contrivances for making aerated waters otherwise than for sale." There are several simple and inexpensive machines on the market which would be quite suitable for small hospitals and infirmaries; and, as the tax on bought syphons of soda-water will add at least 1s. 6d. per dozen to the cost of such syphons, it will clearly be in the interests of economy to install one or other of these contrivances.

### INSURANCE BENEFITS AND SALARIES.

The Southwark Board of Guardians have found themselves in a very serious position as the result of their generous treatment of the staff engaged at their various institutions. It had been the practice to pay full wages to all officers when sick, in addition to which they received the usual benefits under the National Insurance Act, but, as the result of a recent decision of the Local Government Board auditor, they decided to deduct from the full salaries the sums the officers got in the way of sick benefits. The officers, however, have revolted against this decision, and there are threats of strikes amongst certain sections of the medical and nursing staffs if the decison of the board is carried out, in fact, one officer has declined to allow any deductions to be made from his salary in the case of illness. The Public Auditor has laid down the rule that no officer is entitled to make a profit out of the National Insurance Act, as he would do if he was given full pay and received the sick benefit in addition. What the result may be remains to be seen, but in the meantime the officers, to the number of one hundred, have received the permission of the Guardians to discuss the matter.

### THIS WEEK'S DRUG MARKET.

With few exceptions all the price changes are in an upward direction. Quotations for cod-liver oil come much higher from Norway, but buyers of the oil for home consumption still keep off the market; the cause of the recent sharp advance is believed to be that Germany, Russia, and America are buying heavily. The high values of tartaric and citric acids are well maintained for the present. Camphor has an upward tendency in price. Hexamine continues in good demand and prices are advancing. There has been no further change in quotations for bromides. Cocoa butter and saccharin are dearer. Caffeine continues extremely scarce, and although makers' prices are nominally unchanged, holders of small stocks are able to obtain 50 per cent. above makers' quotations. The high value of cocaine is still very firmly maintained. Salol is getting scarcer, and prices have an upward tendency. At the recent public auction of drugs in Mincing Lane, senna-leaves, Cape aloes, and honey fetched higher prices.

